# Long QT Syndrome Unveiled by a Fatal Combination of Medications and Electrolyte Abnormalities

**DOI:** 10.7759/cureus.1581

**Published:** 2017-08-18

**Authors:** Pooja Sethi, Jennifer Treece, Vandana Pai, Chidinma Onweni

**Affiliations:** 1 Department of Cardiology, Quillen College of Medicine, East Tennessee State University; 2 Internal Medicine, Quillen College of Medicine, East Tennessee State University

**Keywords:** long qt syndrome, torsades de pointes, qt interval, implantable cardioverter defibrillator, syncope, seizure

## Abstract

Long QT syndrome (LQTS) can present with syncope and seizure-like activity in the setting of torsades de pointes (TdP) with hemodynamic instability. Electrolyte abnormalities and medications can predispose to TdP in the setting of latent LQTS. An implantable cardioverter defibrillator (ICD) is needed if patients with TdP continue to be symptomatic despite medical treatment. We report a case of a patient who presented with seizures and was found to have prolonged corrected QT interval (QTc). During her admission, she was treated with ondansetron. She went into torsades de pointes and continued to have prolonged QTc. She underwent implantable cardioverter defibrillator (ICD) placement and remains asymptomatic to date.

## Introduction

Long QT syndrome (LQTS) is a congenital condition characterized by prolongation of the QT interval to >440 ms in men and >460 ms in women on electrocardiogram (ECG). Medications and electrolyte abnormalities predispose patients with LQTS to develop torsades de Pointes (TdP), which is a potentially life-threatening polymorphic ventricular tachyarrhythmia [1].

## Case presentation

A 19-year-old woman with no prior past medical history presented to the emergency department with an episode of shaking and seizure-like activity. The episode lasted for five minutes and was followed by a syncopal episode that lasted an additional fifteen minutes. The patient did not have any bowel or bladder incontinence nor did she have tongue biting during this episode. She spontaneously regained consciousness following the 15-minute syncopal episode and experienced confusion immediately following the episode. One week prior, she had experienced a spontaneous abortion. Her pregnancy had been complicated by hyperemesis gravidarum, and her symptoms of nausea and vomiting had persisted following the termination of the pregnancy. She denied any family history of seizure activity or sudden death. At the initial physical examination, her blood pressure was 145/110 mmHg, her heart rate was 117 beats per minute, and her respiratory rate was 16 breaths per minute. On neurological examination, she was alert and oriented, and the strength and tone in all extremities were intact. The remainder of her physical exam was benign. Her labs showed potassium of 2.7 mEq/L and magnesium of 1.4 mg/dL. Her ECG showed a QT interval of 304 milliseconds (ms) with corrected QT interval (QTc) of 550 ms. Unfortunately, we no longer have access to the patient's ECG or telemetry strips.

Her nausea and vomiting were treated with intravenous ondansetron, and her electrolytes (magnesium was 1.4 mg/dL and potassium 2.7 mEq/L) were replaced to maintain magnesium greater than two and potassium four. Her electroencephalogram was normal. She had two subsequent episodes of seizure-like activity while hospitalized. Telemetry monitoring revealed continuous prolonged QT interval and runs of torsades de pointes (TdP) during these episodes. Long QT syndrome (LQTS) was suspected, and ondansetron was implicated for pushing the LQTS to TdP, which was thought to be causing syncope with seizure-like activity in this patient. Ondansetron and all other QT prolonging medicines were discontinued at that time. The patient, however, continued to have episodes of syncope with seizure-like activity and runs of TdP despite being placed on isoproterenol and later lidocaine therapy. Her QT remained prolonged with QT greater than 580 and calculated QT greater than 700 ms. She underwent implantable cardioverter defibrillator (ICD) placement and was discharged. Following placement of the ICD, the patient later became pregnant again and had a normal delivery and remains asymptomatic to this day.

## Discussion

Epidemiology

Prolonged QTc interval may be congenital or acquired. Congenital long QT syndrome results from genetic mutations, occurs in 1:2,000 births, and accounts for approximately 12% of sudden infant death syndrome (SIDS) [1]. Acquired prolonged QTc is most commonly secondary to medications [2]. QT-prolonging medications include ondansetron, azithromycin, amiodarone, as well as many antipsychotic medications, which cause prolongation of the QT segment and thereby increase the risk of development of TdP [3]. Electrolyte abnormalities like hypokalemia or hypomagnesemia when combined with prolonged QT can further increase the risk of developing TdP. The incidence is 1:10,000. In as many as 10-15% of patients with drug-induced TdP, a form of congenital LQTS exists, causing these patients to be more susceptible to drug-induced prolonged QT [4].

Risk factors

The risk factors for the development of TdP include a QTc (corrected) interval greater than 500 ms, a QTc interval increase greater than 60 ms from the value prior to medication initiation, hypomagnesemia, hypokalemia, advanced age, heart failure with reduced ejection fraction, myocardial infarction, bradycardia, and medications that prolong the QTc interval. Medications that prolong the QTc interval include ondansetron, antipsychotics (haloperidol), azithromycin, and anti-arrhythmia medications (amiodarone, dofetilide) [5]. Seventy percent of patients with LQTS are women; the female gender is at higher risk for prolonged QTc interval than males as testosterone has a QTc interval shortening effect.

Clinical features

The symptoms related to TdP are secondary to the rapid heart rate of this arrhythmia, which leads to cardiopulmonary compromise with hypotension and decreased cardiac output. The heart rate of TdP ranges from 160 to 240 beats per minute [6]. TdP presents as palpitations, shortness of breath, dizziness or lightheadedness, and syncopal episodes that may be accompanied with seizure-like activity [5]. The patient in this case had similar symptoms—syncope, tachycardia and seizure. Although it is possible for TdP to be non-sustained and spontaneously resolve, TdP can also devolve into ventricular fibrillation, which can be fatal [5].

Diagnosis

Diagnose QT prolongation by inspecting a patient’s ECG to determine if the QTc is greater than 440 ms for males or greater than 460 ms for females. Development of TdP secondary to prolonged QT can also be visualized on ECG as well as telemetry continuous cardiac monitoring [5].

Our case demonstrates an important type of prolonged QT—congential long QT syndrome. Our patient is very young with no other health issues and not on any medications that predispose or cause QT prolongation. Because long QT syndrome (LQTS) is a cardiac arrhythmia that predisposes otherwise healthy young patients to developing torsades de pointes-induced-syncope, seizures, and sudden death, detection of a genetic predisposition for LQTS is important to mitigate its effects on the patients’ quality of life and life expectancy. The three genes that account for approximately 75% of patients with LQTS include genes KCNH2, KCNQ1, and SCN5A. It is recommended that genetic testing be performed on patients with persistently prolonged QT intervals despite being asymptomatic and on patients for whom the diagnosis of LQTS is highly suspected due to the patients’ clinical presentation [7]. Genetic testing for LQTS of family members of patients with confirmed LQTS is controversial as only 75% can be detected through searching for the three known genes related to LQTS, and the presence of a detected gene does not necessarily mean that the individual will develop LQTS due to differing levels of penetrance within a poputation [8]. Genetic testing is a definitive path to diagnose LQTS. This was not performed on this patient. Since her QT interval remained prolonged and she had episodes of Tdp, treatment with an ICD was indicated.

Treatment

For patients who are hemodynamically stable, TdP is treated by discontinuing medications that prolong the QT interval and replacing the potassium and magnesium to maintain a potassium level greater than four and magnesium level greater than two [6]. Magnesium sulfate 1-2 grams intravenous should be given acutely in any patient with TdP, as this rhythm can cause sudden cardiac death by rapidly devolving into ventricular fibrillation, which is a life-threatening arrhythmia [5]. For patients who are hemodynamically unstable (hypotensive, unresponsive) and have TdP, the treatment is defibrillation [5]. In patients with recurrent TdP, increasing the heart rate through the use of isoproterenol will shorten the QT interval and therefore decrease this arrhythmia. Long term maintenance therapy involves using beta blockers to prevent the development of tachyarrhythmias [9]. Implantable cardiac defibrillator (ICD) is indicated in patients who continue to have syncopal episodes despite beta blockade and in patients with very prolonged QTc intervals of greater than 550 ms [10].

## Conclusions

LQTS is a congenital condition characterized by prolongation of the QT interval to greater than 440 ms in men and greater than 460 ms in women. Medications like ondansetron in our patient and electrolyte abnormalities like hypokalemia and hypomagnesemia predispose patients with LQTS to TdP. The syndrome has a female predominance with 70% of patients with LQTS being women. The incidence is 1:10,000, but it largely remains undiagnosed until an offending drug or electrolyte abnormality unmasks the condition. It frequently presents as seizure and syncopal episodes. This patient did not have a history of structural heart disease or family history of sudden cardiac death and was not on any medications or substances other than those prescribed. TdP was secondary to underlying LQTS that was unmasked through the use of QT prolonging medications in conjunction with hypokalemia and hypomagnesemia. Beta blockers are the treatment of choice. ICD is indicated if patients continue to have symptoms and TdP while on isoproterenol or if they have a very prolonged QTc greater than 500 ms, and the patient in this case met both criteria for IDC placement.
